# Comparison of Antibody Repertoires Produced by HIV-1 Infection, Other Chronic and Acute Infections, and Systemic Autoimmune Disease

**DOI:** 10.1371/journal.pone.0016857

**Published:** 2011-03-30

**Authors:** Felix Breden, Christa Lepik, Nancy S. Longo, Marinieve Montero, Peter E. Lipsky, Jamie K. Scott

**Affiliations:** 1 Department of Biological Sciences, Simon Fraser University, Burnaby, British Columbia, Canada; 2 Department of Molecular Biology and Biochemistry, Simon Fraser University, Burnaby, British Columbia, Canada; 3 Repertoire Analysis Group, Autoimmunity Branch, National Institute of Arthritis and Musculoskeletal and Skin Diseases, National Institutes of Health, Bethesda, Maryland, United States of America; 4 Faculty of Health Sciences, Simon Fraser University, Burnaby, British Columbia, Canada; Fundació Institut Germans Trias i Pujol; Universitat Autònoma de Barcelona CibeRES, Spain

## Abstract

**Background:**

Antibodies (**Abs**) produced during HIV-1 infection rarely neutralize a broad range of viral isolates; only eight broadly-neutralizing (**bNt**) monoclonal (**M**)Abs have been isolated. Yet, to be effective, an HIV-1 vaccine may have to elicit the essential features of these MAbs. The V genes of all of these bNt MAbs are highly somatically mutated, and the V_H_ genes of five of them encode a long (≥20 aa) third complementarity-determining region (**CDR-H3**). This led us to question whether long CDR-H3s and high levels of somatic mutation (**SM**) are a preferred feature of anti-HIV bNt MAbs, or if other adaptive immune responses elicit them in general.

**Methodology and Principal Findings:**

We assembled a V_H_-gene sequence database from over 700 human MAbs of known antigen specificity isolated from chronic (viral) infections (**ChI**), acute (bacterial and viral) infections (**AcI**), and systemic autoimmune diseases (**SAD**), and compared their CDR-H3 length, number of SMs and germline V_H_-gene usage. We found that anti-HIV Abs, regardless of their neutralization breadth, tended to have long CDR-H3s and high numbers of SMs. However, these features were also common among Abs associated with other chronic viral infections. In contrast, Abs from acute viral infections (but not bacterial infections) tended to have relatively short CDR-H3s and a low number of SMs, whereas SAD Abs were generally intermediate in CDR-H3 length and number of SMs. Analysis of V_H_ gene usage showed that ChI Abs also tended to favor distal germline V_H_-genes (particularly V_H_1-69), especially in Abs bearing long CDR-H3s.

**Conclusions and Significance:**

The striking difference between the Abs produced during chronic *vs.* acute viral infection suggests that Abs bearing long CDR-H3s, high levels of SM and V_H_1-69 gene usage may be preferentially selected during persistent infection.

## Introduction

A highly diverse repertoire of antibodies (**Abs**) is a prerequisite for the adaptive immune system to recognize a vast array of antigens (**Ags**) and distinguish self from non-self. Three processes contribute to the production of this diverse repertoire: (i) somatic recombination of germline V, D and J genes, (ii) addition and deletion of nucleotides at the V-D, D-J, and V-J junctions, and (iii) somatic hypermutation after Ag stimulation [1], [2]. The third complementarity-determining region of the Ab heavy chain (**CDR-H3**) is encoded by the D_H_ gene, parts of the V_H_ and J_H_ genes, and nucleotides added at the junctions between these; it is the most variable region in the Ab, and typically is central to contact with cognate Ag [3].

A major goal for an HIV vaccine is to elicit Abs that neutralize a broad range of HIV-1 primary isolates. To this end, efforts have been made to identify and use broadly (**b**) neutralizing (**Nt**) monoclonal (**M**) Abs with this activity for epitope-targeted vaccine design [4]. The bNt MAbs identified so far are rare and most of them bear unusually long CDR-H3s. Despite intensive effort, only eight bNt MAbs have been discovered (b12, 2F5, 4E10, 2G12, 447-52D, PG9/PG16, VRC01/02, and HJ16 [5], [6], [7], [8], [9]; 5 of which bear CDR-H3s of 20 aa or more, based on the IMGT numbering system). Consistent with this, most HIV-1-infected individuals produce strong strain-specific Nt Ab responses against HIV-1 envelope (**Env**) soon after initial infection; yet rarely do they develop broad neutralization [10], [11], and then only after a year or more [12].

While high levels of SM have been noted for all bNt MAbs, starting with Kunert *et al*. [13], a number of authors have proposed a connection between the length of the CDR-H3 region, and the broad neutralization of these MAbs [5], [7], [14], [15], [16], [17], [18], [19], [20], [21]. Mutagenesis experiments and/or X-ray crystal structures of Fab bound to protein or peptide Ag have implicated their CDR-H3s as being required for neutralization: b12 [15], [22], 2F5 [16], [23], [24], 447-52D [17], and 4E10 [18], [25]. This has been observed even in cases in which CDR-H3 appears to make minimal or no contact with envelope protein Ag [16], [23]. It has been speculated that in these cases, CDR-H3 may contact other sites on HIV-1, such as the viral membrane [16], [18], [19], [20], [24], [25], [26]. Nevertheless, it is not clear whether long CDR-H3s are required, in general, for broad neutralization; certainly the exceptions, MAbs 2G12 and VRC01/02, disprove this as an absolute rule for broad Nt activity.

While it is generally acknowledged that high levels of SM are produced by T-cell driven processes in germinal centers, the conditions under which long CDR-H3 Abs appear in adaptive immune responses are less well understood, and this could help explain the origin of bNt Abs during HIV infection. Importantly, the long CDR-H3s have been associated with anti-protein Abs [27] and anti-viral Abs [28]. Long CDR-H3s found among polyreactive natural Abs [29], [30] and autoreactive Abs produced by naïve B cells in SLE patients [31] do not carry SMs; yet, those found among the memory B cells in healthy people do [32]. However, analyses to directly associate different types of adaptive immune response with CDR-H3 length have not been reported. Our purpose was to identify the circumstances under which Abs bearing some of the features of bNt MAbs (*viz*., long CDR-H3s and high levels of SM) appear during adaptive immune responses in humans.

As an initial approach, we examined heavy chain variable (**V_H_**) genes expressed by individuals undergoing Ag-specific immune responses, by compiling a database of expressed human V_H_ genes of MAbs for which the Ag specificity of the MAb was known. The MAbs were taken from individuals with chronic infections (**ChI**), acute infections (**AcI**), or following immunization (included with AcI MAbs), and systemic autoimmune diseases (**SAD**). CDR-H3 length, level of SM, and V_H_-gene usage were compared among MAbs with specificity against self *vs.* non-self Ag, with specificity against protein *vs.* non-protein Ag, and/or from different conditions (ChI MAbs, SAD MAbs and AcI MAbs).

Both long CDR-H3s and SMs were strongly associated with protein Ag. Long CDR-H3s were at their highest frequency among ChI MAbs, and less so among SAD and AcI MAbs, whereas SMs were more prevalent in ChI and SAD anti-protein Abs and greatly reduced among anti-protein AcI Abs. Both ChI and AcI Abs tended to use distal V_H_ genes; the use of V_H_1-69 was especially high among anti-HIV Abs, and was associated with high levels of SM and long CDR-H3s. The picture emerging from this analysis is that Abs bearing high numbers of SMs, long CDR-H3s and the distal gene V_H_1-69 appear to be selected in chronic *vs*. acute viral infections. Thus different biological processes, and perhaps different B-cell subsets, such as marginal zone *vs.* conventional B2 B cells [33], [34], (see Baumgarth [35] for review) could be involved in the earlier *vs*. later stages of viral infection, respectively.

## Results

### CDR-H3 length of expressed V_H_ genes in adaptive Ab responses

**Figure 1:**
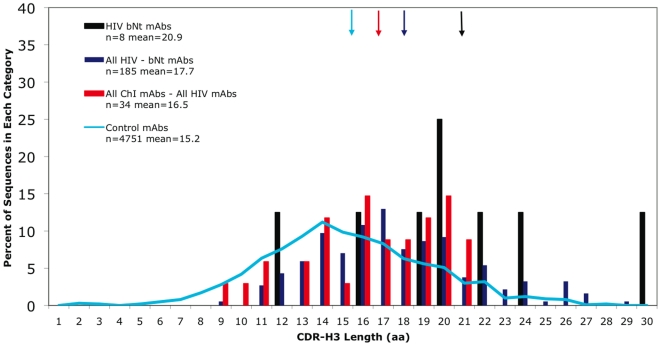
Distribution of CDR-H3 length for HIV Mabs. HIV Mabs are divided into three categories: bNt HIV MAbs, all HIV MAbs with the bNt HIV MAbs removed (*i.e.,* non-bNt HIV MAbs), and ChI MAbs with the HIV MAbs removed (*i.e*., non-HIV ChI MAbs). Arrows indicate the mean for each category. Distribution of CDR-H3 length for more than 4000 Abs compiled from IMGT and Kabat databases [63] is included in Figs. 1–3, as a control comparison (blue line). The average CDR-H3 length of the 425 Ag-specific MAbs was 16.3 aa, which is higher than the 15.2-aa mean reported for 4751 expressed V_H_ sequences compiled from the Kabat and IMGT databases by Zemlin *et al.* [63]; this may reflect that our data set included a higher proportion of ChI Abs, both HIV and non-HIV.

**Table 1** summarizes the analysis of expressed V_H_ genes for several MAb categories with regard to CDR-H3 length, number of SMs relative to the predicted germline V_H_ gene, and the distance in the IgH locus between an Ab's germline V_H_ gene and the V_H_6-1 gene, the V_H_ gene closest to the D_H_ region. As expected, the bNt HIV MAbs had the longest CDR-H3s with an average length of 20.9 aa. **Fig. 1** compares the CDR-H3 length distributions for the bNt HIV MAbs, the non-bNt HIV MAbs (*i.e*., excluding the bNt HIV MAbs), and the remaining ChI MAbs (excluding all HIV MAbs). It shows that both the bNt and non-bNt HIV MAbs have long CDR-H3s; the difference between these two MAb groups was only marginally significant (**Table 1A**; unadjusted p  =  0.0501), and not significant compared to the Bonferroni corrected value of 0.0018. Thus, CDR-H3 length does not appear to be restricted to broad neutralization. In addition, the CDR-H3s of all of the anti-protein HIV MAbs (mean 17.8 aa) were not significantly longer than those of non-HIV ChI MAbs (16.5 aa). Although our data set has only 34 non-HIV ChI MAbs, 12 of these have CDR-H3s of 19 aa or longer (**Fig. 1**), placing them in the upper quartile of the 427 Ag-specific MAbs. Thus, long CDR-H3s were associated with all types of ChI MAb, including the anti-HIV MAbs.

**Table 1:** Comparison of CDR-H3 length, SM and distance from D_H_ of V_H_ genes among MAbs of known Ag specificity associated with HIV infection (Table 1A), chronic infection (ChI), acute infection (AcI) or systemic autoimmune disease (SAD) (Table 1B), and self *vs.* non-self (Table 1C).

MAb Type^a^	# MAbs	CDR-H3 (aa)	*p* b	# MAbs	V_H_ SM	*p*	# MAbs	V_H_ Distance^c^	*p*
**All**	427	16.3	--	426	21.0	--	413	445	--
**A. HIV bNt**	8	20.9	0.0501	8	53.3	0.0024	8	303	0.0541
**HIV Non-bNt**	185	17.7		184	27.3		167	508	
**HIV/P**	192	17.8	0.2095	191	28.2	0.0209	181	503	0.0952
**Non-HIV/P**	34	16.5		34	21.0		32	440	
**B. ChI**	227	17.6	**0.0001 A**	226	27.3	**0.0001 A**	214	492	**0.0001**
**AcI**	113	14.7	B	113	10.9	B	112	414	B
**SAD**	87	15.1	B	87	17.9	B	86	369	B
**ChI/NP**	1	16.0	0.5140	1	60.0	0.0836	1	286	0.6627
**AcI/NP**	43	14.0		43	15.5		43	357	
**SAD/NP**	60	15.0		60	13.2		60	379	
**ChI/P**	226	17.6	**0.0001 A**	225	27.1	**0.0001 A**	204	493	**0.0018 A**
**AcI/P**	70	15.1	B	70	7.9	B	69	450	A/B
**SAD/P**	27	15.4	B	27	28.5	A	26	346	B
**C. Self**	87	15.1	0.0031	87	17.9	0.0370	86	369	**0.0003**
**Non-self**	340	16.6		339	21.8		327	466	
**NP**	104	14.6	**0.0001**	104	14.6	**0.0001**	104	369	**0.0001**
**P**	323	16.9		322	23.1		309	471	
**Self/NP**	60	15.0	0.3519	60	13.2	**0.0001**	60	379	0.6206
**Self/P**	27	15.4		27	28.5		26	346	
**Non-self/NP**	44	14.0	**0.0001**	44	16.6	0.0148	44	355	**0.0001**
**Non-self/P**	296	17.0		295	22.6		283	482	

a MAbs are categorized by Ag type and condition: bNt, broadly neutralizing; NP, non-protein; P, protein; SAD, autoimmune disease; ChI, chronic infection; AcI, acute infection and vaccination; Self  =  SAD MAbs; non-Self  =  ChI plus AcI MAbs.

b Differences between means were analyzed by the Kruskall-Walis Test (PROC NPAR1WAY, SAS), and the uncorrected p values are shown. This analysis involved testing 9 groupings of MAbs (*e.g*. HIV bNt *vs.* HIV Non-bNt) for 3 parameters (CDR-H3 length, SM, and distance of V_H_ gene from V_H_6-1), or 27 tests of heterogeneity among sets of MAbs. Therefore the critical value adjusted for multiple tests (*i.e.*, bonferroni correction, see Materials and Methods) was 0.05/27  = .0019; all p values satisfying this corrected level of significance are shown in bold). When the Kruskall-Wallis test showed significant heterogeneity among 3 categories of MAb being compared (*i.e.*, ChI, AcI and SAD), we used parametric, *a posteriori* Tukey pair-wise comparisons (PROC GLM, SAS) to determine which sets of MAbs were contributing to the heterogeneity. For this Tukey *a posteriori* test, letters denote conditions that differ at the 5% level. For example, ChI, AcI and SAD Abs were heterogeneous for CDR-H3 length at the p<0.0001 level according to the Kruskall-Wallis test, and the Tukey *a posteriori* test grouped AcI and SAD Abs together (letter B), but different from ChI Abs (letter A).

c Distance between an Ab's germline V_H_ gene and the V_H_6-1 gene, the V_H_ gene closest to the D_H_ region.

**Figure 2:**
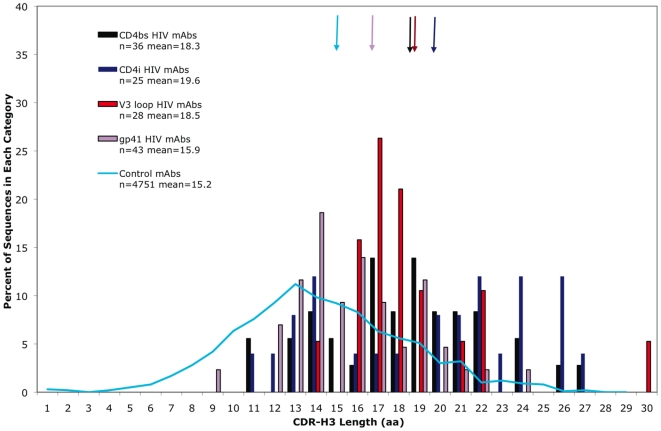
Distribution of CDR-H3 length for HIV MAbs against the different sites on Env. Env sites are categorized as: CD4bs, CD4i, V3 loop, gp41, and control MAbs (blue line) [63]. Arrows indicate the mean for each category.

**Fig. 2** shows the distribution CDR-H3 length for anti-HIV MAbs, partitioned according the region of Env bound; CDR-H3 length was longest for MAbs against the CD4i site (19.6 aa), intermediate for those against the V3 loop (18.5 aa) and CD4bs (18.3 aa), and shortest for the anti-gp41 MAbs (15.9 aa). These distributions were significantly different (χ^2^ test, p<0.05, PROC FREQ, SAS), indicating that, while the anti-HIV MAbs as a whole bear long CDR-H3s, epitope specificity also shapes CDR-H3 length.

**Figure 3:**
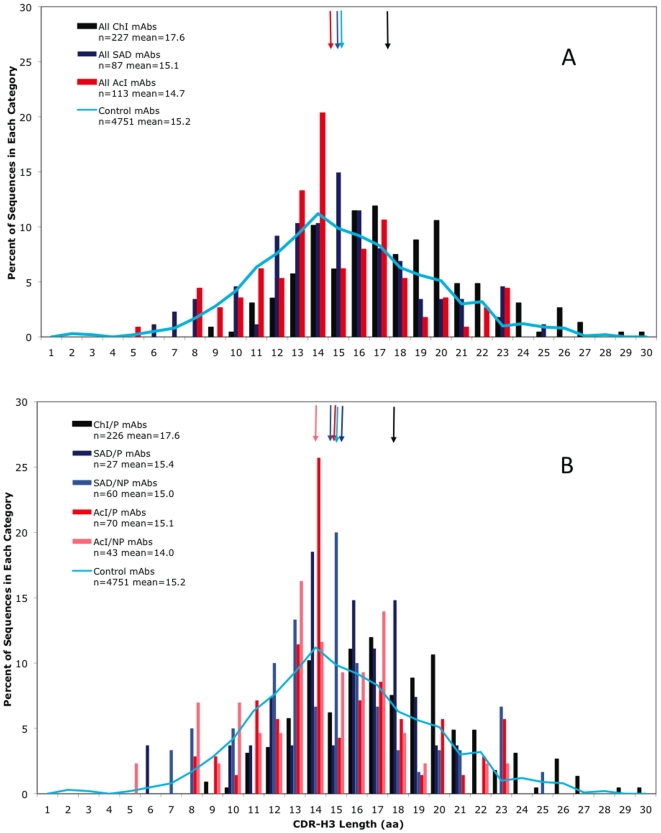
Distribution of CDR-H3 length for different types of immune response and Ag type. A. Distribution of CDR-H3 length for ChI, SAD, and AcI MAbs. B. Distribution of CDR-H3 length for ChI anti-protein, SAD anti-protein, AcI anti-protein, SAD anti-non-protein, and AcI anti-non-protein MAbs. Arrows indicate the means for each category. Control MAbs (blue line) are included for comparison [63].

The observation that long CDR-H3s are associated with chronic viral infections in general led us to compare MAbs from such infections to those from other immune responses, namely SAD and AcI. Categorization of MAbs by these types of immune response showed that the average length of the ChI MAbs (17.6 aa, **Table 1B** and **Fig. 3A**) was most different from the AcI MAbs (14.7 aa), with the SAD MAbs being intermediate (15.1 aa). This trend, of the AcI MAbs being the most different from the ChI MAbs, persisted when the categories were further divided into Abs against protein *vs.* non-protein Ags (**Fig. 3B** and **Table 1B**). For example, among anti-protein MAbs, those from the ChI group had significantly longer CDR-H3s than did those from the AcI or SAD groups (p<0.0001). As virtually all of the ChI Abs (except bNt MAb 2G12) and all of the anti-protein AcI Abs are anti-viral, it is striking that a large difference in CDR-H3 length exists between the Abs elicited by the two types of viral infection. In addition, anti-protein MAbs had significantly longer CDR-H3s than MAbs against non-protein Ags (p<0.0001, **Table 1C**), even with the Bonferroni correction; while the difference between self and non-self Abs was not as great (p<0.005, not significant with the Bonferroni correction). Thus autoimmune status (self *vs*. non-self) had a much lower effect on CDR-H3 length than did protein *vs.* non-protein Ag.

### SM of expressed V_H_ genes in adaptive Ab responses

That long CDR-H3s were found mainly among anti-protein Abs suggests that Abs with long CDR-H3s are selected by protein Ag, and hence, by T-cell-driven processes; such responses typically occur in germinal centers and involve SMs introduced by activation-induced cytidine deaminase (**AID**) [36]. Predicting that SMs should increase among Abs bearing long CDR-H3s, we compared the patterns of SM for the same categories of MAb as for CDR-H3 length in **Table 1**. For many comparisons, the patterns observed for SMs paralleled those observed for CDR-H3 length. For example, the eight bNt HIV MAbs had both the highest average CDRH3 length and the highest average level of SM in V_H_ (mean 53.3). Given the small number of bNt MAbs, these were significantly longer than the non-bNt HIV MAbs (mean 27.3, p = 0.0024), but not when the Bonferroni correction for multiple tests was used. In comparing MAbs from the different types of immune response, the ChI MAbs (SM mean 27.3) were, again, most different from the AcI MAbs (mean 10.9), with the SAD MAbs being intermediate (mean 17.9). The SAD and AcI MAbs showed opposite patterns when they were partitioned according to protein *vs*. non-protein Ag; for SAD, the SM level among anti-protein MAbs was higher than that of MAbs against non-protein Ags, whereas for AcI MAbs, the level of SM was lower for protein *vs*. non-protein Ags. The average number of SMs for AcI MAbs against non-protein Ags, which were mainly against streptococcal capsular polysaccharide, was 15.5, intermediate between ChI and SAD non-protein MAbs. However, the level of SM was extremely low for AcI MAbs against protein Ags (mean 7.9), many of which were against rotavirus (more than 40%). This reduction in SMs among the anti-protein AcI MAbs was not restricted to anti-rotavirus MAbs, as when they were excluded from the AcI MAb category, the anti-protein AcI MAbs still had a low number of SMs (mean 7.3). In summary, there was a significant difference between Abs from chronic and acute viral infections, with the latter consistently having much shorter CDR-H3s and far fewer SMs. Little difference in SMs was observed between anti-protein Abs produced by chronic processes (ChI and SAD); both had long CDR-H3s and high levels of SM, and both involve persistent exposure to Ag.

To further analyze the relationship between CDR-H3 length and levels of SM, the MAb dataset was divided into quartiles according to CDR-H3 length, with the shortest quartile (S) having lengths of 13 aa or less, the longest quartile (L) 19 aa or more, and the two middle quartiles comprising the M class (between 14 and 18 aa inclusive, **Table 2**). While the differences in SM levels observed in **Table 1B** among the disease conditions or types of Ag specificities generally held across the corresponding quartiles in **Table 2**, some differences were observed. **Table 2B** shows little difference in the number of SMs between the anti-protein Abs for all disease categories and little difference in SM levels between the quartiles for medium-length and the longest CDR-H3s; however, the short CDR-H3 quartile tended to have lower levels of SM than did the longer two groups. Furthermore, the trend for MAbs against non-protein Ags was reversed: MAbs in the shortest CDR-H3 quartile tended to have the highest SM levels for both the SAD and AcI categories. Although long CDR-H3 MAbs against non-protein Ags are uncommon, their SM level was lower than that of their short CDR-H3 counterparts. This is consistent with the hypothesis that MAbs against non-protein Ags (even those with long CDR-H3s) may derive from different B-cell subsets and/or different immune processes than the MAbs against protein Ags.

**Table 2:** Somatic mutations and V_H_-gene distance of MAbs grouped according to short (< = 13aa), medium (14-18 aa) or long (> = 19aa) CDR-H3 length.

MAb Type^a^	V_H_ SM			V_H_ Distance^b^		
	S	M	L	S	M	L
**All**	16.5	21.8	22.9	353	466	488
**A. HIV bNt**	NA^c^	76.5 (2)^d^	45.5 (6)	NA	286 (2)	320 (6)
**B. ChI**	24.1	27.9	26.1	327	498	539
**AcI**	10.6	11.5	8.9 (15)	401	448	322 (15)
**SAD**	16.5	18.9	17.7 (14)	299	405	394 (14)
**ChI/NP**	NA	60.0 (1)	NA	NA	286 (1)	NA
**ChI/P**	24.1	28.2	26.9	333	504	534
**HIV/NP**	NA	60.0 (1)	NA	NA	286 (1)	NA
**HIV/P**	25.3	29.7	27.3	334	517	537
**Not HIV/NP**	NA	NA	NA	NA	NA	NA
**Not HIV/P**	19.0 (6)	19.6 (16)	24.0 (12)	328 (6)	424 (14)	516 (12)
**AcI/NP**	15.8 (19)	15.6 (21)	14.3 (3)	329 (19)	400 (21)	229 (3)
**AcI/P**	6.1 (22)	9.2	7.6 (12)	466 (21)	476	346 (12)
**SAD/NP**	15.0 (22)	11.9	12.8 (10)	327 (22)	402	429 (10)
**SAD/P**	22.0 (6)	30.4 (17)	30.0 (4)	197 (6)	411 (16)	307 (4)
**C. Self**	16.5	18.9	17.7 (14)	299	405	394 (14)
**Non-self**	16.4	22.6	24.3	375	483	502
**NP**	15.4	14.4	13.2 (13)	328	399	382 (13)
**P**	17.2	24.1	24.8	370	488	502
**Self/NP**	15.0 (22)	11.9	12.8 (10)	327 (22)	402	429 (10)
**Self/P**	22.0 (6)	30.4 (17)	30.0 (4)	197 (6)	411 (16)	307 (4)
**Non-self/NP**	15.8 (19)	17.6 (22)	14.3 (3)	329 (19)	395 (22)	229 (3)
**Non-self/P**	16.6	23.3	24.6	392	497	510

a MAbs are categorized by Ag type and condition: Nt, broadly neutralizing; NP, non-protein; P, protein; SAD, autoimmune disease; ChI, chronic infection; AcI, acute infection and vaccination; Self  =  SAD MAbs; non-Self  =  ChI plus AcI MAbs.

b Distance between an Ab's germline V_H_ gene and the V_H_6-1 gene, the V_H_ gene closest to the D_H_ region.

c NA, Not available; *i.e*., there were no MAbs of this type in the database.

d Sample sizes**<**25 are given in parentheses.

### Germline V_H_-gene usage in adaptive Ab responses

Early reports of V_H_ gene family usage in anti-HIV Abs reported that V_H_3 was over-utilized and V_H_4 was under-utilized compared to the naïve repertoire [37], [38]. Given that our dataset includes Abs of known Ag specificity for several disease conditions, we compared the use of V-gene families and of the specific gene V_H_1-69 among these conditions (**Table 3** and **Figure S1**). **Table 3** shows that there were significant differences when the proportions were tested across each gene family. The proportion of anti-HIV MAbs that use family V_H_3 genes (29%) was lower than that for SAD MAbs (52%) or AcI MAbs (53%). Concomitantly, use of family V_H_1 increased for HIV MAbs (38% for HIV compared to 23% for SAD MAbs and 22% for AcI MAbs), whereas the proportion of MAbs using family V_H_4 was not significantly different among the major categories, ranging from 18 to 25%. Thus, HIV infection was related to an increase in V_H_1 and decrease in V_H_3 gene usage that was not apparent for ChI Abs (perhaps because this sample size is small) or other conditions.

**Table 3:** V_H_ gene family and V_H_1-69 gene usage among 4 types of MAbs.

Gene Family	Condition				p^a^
	% HIV^b^	% CHI, not HIV	% SAD	% AcI	
**A.** c **V_H_-1**	39 (76)^c^	21 (7)	23 (20)	22 (25)	**
**V_H_-2**	2 (3)	0 (0)	1 (1)	0 (0)	NS
**V_H_-3**	29 (56)	44(15)	52 (45)	53 (60)	**
**V_H_-4**	24 (47)	26 (9)	18 (16)	23 (26)	NS
**V_H_-5**	6 (11)	9 (3)	5 (4)	1 (1)	NS
**V_H_-6**	0 (0)	0 (0)	1 (1)	1 (1)	NS
**Total**	100 (193)	100 (34)	100 (87)	100 (113)	
**Specific Gene**					
**B.** d **V_H_1-69**	22 (43)	12 (4)	6 (5)	3 (3)	**

a NS, Not significant;

**p<0.001.

b For each V_H_ gene family (row), the proportion of MAbs using that gene family was tested for heterogeneity among the 4 types of MAbs (columns) using a 4×2 contingency χ^2^ test (http://www.physics.csbsju.edu/stats/contingency_NROW_NCOLUMN_form.html).

c Data are presented as per cent followed by number of MAbs in parentheses.

d Proportion of MAbs using the V_H_1-69 gene was also tested for heterogeneity among the four sets of MAbs.

We were particularly interested in usage patterns for the V_H_1-69 germline gene, as it is not commonly used in the naïve repertoire (*e.g.*, Wardemann *et al.* 2003 [39]), but is characteristic of Ab repertoires in several disease states (see Discussion). As shown in **Table 3**, analysis of the 193 HIV MAbs having verifiable germline V_H_ genes revealed that 43 (22%) used V_H_1-69, whereas only five of 87 (6%) SAD MAbs and three of 113 (3%) AcI MAbs used this V_H_ gene; V_H_1-69 usage by (non-HIV) ChI MAbs was intermediate, being four of 34 (12%). Thus, HIV MAbs, and, perhaps, ChI MAbs use V_H_1-69 at a higher frequency than do SAD or AcI MAbs.

Given this distinct difference, we directly compared the features of MAbs that use V_H_1-69 to those that do not (**Table 4**). Among ChI MAbs, those that used V_H_1-69 had significantly longer CDR-H3s (means of 20.1 *vs.* 17.0, P<0.001), whereas their SMs were not significantly different. Very few SAD or AcI MAbs used V_H_1-69, so no statistical comparisons could be made within those groups. Among MAbs that use V_H_ genes other than V_H_1-69, the patterns among the disease categories of ChI, SAD and AcI were similar to those observed in the full data set; the CDR-H3 length of ChI MAbs remained significantly longer than those of SAD and AcI MAbs, and the SMs of all three groups were different. Thus, ChI Abs encoded by V_H_1-69 appear to have longer CDR-H3s but not more SMs than their counterparts that do not use this germline gene.

**Table 4:** Association between V_H_1-69 gene usage, CDR-H3 length and number of somatic mutations.

Condition	CDR-H3 length			V_H_ gene SM		
	V_H_1-69	Not V_H_1-69	Tt	V_H_1-69	Not V_H_1-69	Tt^b^
**ChI**	20.1 (47)**^a^	17.0 (180)	A^b^	27.6 (47) NS	27.2 (179)	A
**AcI**	15.3 (3)	14.7 (110)	B	14.7 (3)	10.7 (110)	B
**SAD**	15.2 (5)	15.1 (82)	B	31.2 (5)	17.1 (82)	C

a Mean (# of MAbs). CDR-H3 length and SM were tested between ChI MAbs using V_H_1-69 and those that do not; CDR-H3 length was significantly different (p<0.001), while the average number of SMs was not different (NS). Statistical comparisons were not made between SAD and AcI MAbs due to the low numbers of those MAbs using V_H_1-69.

b Tt, Tukey test. Letters denote conditions (rows) that differ at the 5% level in *a posteriori* comparisons among MAbs not using V_H_1-69. Again, statistical comparisons were made only for those not using V_H_1-69, due to the low numbers of SAD and AcI MAbs that used V_H_1-69.

V_H_1-69 is a fairly distal gene, being approximately 764 Kb from V_H_6-1 [40]; only five of the approximately 40 functional V_H_-genes are more distal. Thus we wondered if the use of V_H_1-69 among Abs bearing long CDR-H3s in HIV infection could be part of a larger trend toward using distal genes in ChI. **Table 1A** shows that ChI MAbs use the most distal V_H_ genes, consistent with this class having the longest CDR-H3s and the highest frequency of SMs. However, inconsistent with CDR-H3 length and SMs, anti-protein (anti-viral) AcI Abs had intermediate V_H_ gene distances whereas SAD Abs had the most proximal ones. Importantly, the AcI MAbs, only three of which use V_H_1-69, used distal genes overall; the average distance for 69 anti-protein (antiviral) AcI MAbs is 450 kb, suggesting that distal genes besides V_H_1-69 may be selected in viral infections of all types.

**Table 2** further analyzes the relationship between CDR-H3 length and V_H_ gene distance, dividing the MAbs into short, medium and long. Table 2 shows that the pattern is also not consistent within disease condition. Analyzing CDR-H3 length by quartile, the trend between CDR-H3 length and V_H_ gene distance held for ChI and SAD but not for AcI Abs. The medium and long quartiles of ChI and SAD MAbs used the most distal V_H_ genes, whereas they were used by the short and medium quartiles of anti-protein AcI MAbs. Thus, within the viral infection groups, and following similar trends for SM, distal gene usage was related to CDR-H3 length for the ChI, but not the AcI, MAbs.

## Discussion

The primary motivation for this analysis was to determine if other types of Ab share features with the bNt HIV MAbs, which might help explain the rarity of bNt MAbs in HIV-1 infection. We found that the bNt MAbs most closely resemble the other anti-HIV MAbs, and ChI MAbs as a group, in being enriched for long CDR-H3s and high numbers of SMs; this indicates that these features are not limited to broad neutralization, but appear to be common characteristics of the Abs involved in chronic viral infections. That all anti-HIV MAbs, including the bNt MAbs, share similar features indicates that unusual immunological processes, such as breaking of tolerance [19], are probably not responsible for the rarity of the bNt Abs during chronic HIV infection. Instead, processes involved in chronic viral infections in general may be at play in shaping the repertoire of Abs available for selection after viral persistence and/or multiple rounds of viral escape; such processes are probably linked to broadening of the Ab response beyond those involved in the response to initial infection [41]. Our results are consistent with the view that Abs having the features of the bNt MAbs are not rare, but arise as a result of chronic viral infection.

Strikingly, the Abs from acute viral infections (anti-protein AcI Abs) had significantly shorter CDR-H3s and lower numbers of SMs than did the ChI Abs. In addition, while both types of Ab tended to use distal V_H_ genes, Abs from acute viral infections bearing long CDR-H3s tended not to use the most distal V_H_ genes, nor did they use V_H_1-69 to the same extent as the ChI (and HIV) Abs. We speculate that, if the Abs involved in acute viral infections reflect those produced during the early phases of chronic viral infection, a shift in expressed V_H_ gene composition (*i.e.,* CDR-H3 length and V_H_ gene usage) must occur over time, along with an increase in SMs.

High SMs, but not long CDR-H3s nor use of distal V_H_ genes, were also found among SAD-related anti-protein MAbs. This lack of shared features between the SAD and bNt MAbs (or ChI MAbs in general) suggests that the bNt MAbs against HIV are probably not drawn from an initial pool of autoimmune B cells bearing long CDR-H3s, as previously hypothesized [19], [20], [42]; were that the case, then the bNt MAbs would be expected to be similar to the SAD MAbs but not the ChI ones. Clearly, Abs having the features of the bNt MAbs are not rare, and are routinely produced during ChIs. Thus, it seems more likely that the rarity of the bNt HIV Abs results from the cryptic, flexible and/or transient nature of conserved epitopes on the neutralization-competent structure of HIV Env; such epitopes are not immunodominant on the virus, nor on the envelope “debris” shed by infected cells, and as such, multiple rounds of viral escape are likely required before the immune system can mount an effective Ab response against them.

This study is to our knowledge the first to explicitly compare gene family usage in MAbs from HIV with those from other types of immune response. We observed a bias toward family V_H_1 and against family V_H_3 genes in the HIV and other ChI MAbs. The increased usage of family V_H_1 agrees with Scheid *et al.* [43]; and removal of the large number of Abs from Scheid *et al.* did not affect this conclusion (analysis not shown). A deficit in family V_H_3 usage associated with HIV infection has been reported in several studies [37], [38], [44], [45], [46]. This deficit is consistent with the suggestion that HIV-1 gp120 acts as a superAg that specifically deletes B cells bearing Abs encoded by genes from family V_H_3 [47], [48]. In addition, and in contrast to some previous findings [45], we did not observe over-utilization of the V_H_4 family, which remained mostly constant across all MAb categories.

We observed an overabundance of V_H_1-69 gene usage in the HIV MAbs and among ChI MAbs in general, compared to the naïve repertoire reported from other studies [39], and to the other MAb categories in our database. Our results extend the observations of Huang *et al.* [49], who noted that nine of twelve MAbs against the CD4i site of gp120, used V_H_1-69, and those of Gorny *et al.* [46], who showed that MAbs against all HIV Env epitopes, except the V3 loop, are enriched for V_H_1-69 usage. In addition, three studies have noted almost exclusive use of V_H_1-69 among cross-protective MAbs against influenza virus [50], [51], [52].

Both AcI and ChI MAbs tended to use distal V_H_ genes, but only in the latter group were long CDR-H3s present in Abs encoded by distal V_H_ genes, which tended to be V_H_1-69. Three mechanisms can produce long CDR-H3s: (i) longer V_H_, D_H_, or J_H_ genes can be used preferentially, (ii) CDR-H3 can be lengthened by insertions induced by activation-induced cytidine deaminase [9], [53], and (iii) secondary rearrangement (or receptor editing or revision [54], [55]) can result in N and P additions at the N1 junction. Secondary V_H_-gene rearrangement necessarily involves the use of distal V_H_ and J_H_ genes, because once a V_H_ gene is somatically recombined with a D_H_ gene (*i.e*., after primary V-D-J rearrangement), only genes more distal to the D_H_ region are available for further joining. The features of the ChI Abs alone are consistent with secondary rearrangement model, in both using distal V_H_ genes and having long CDR-H3s for the same Ab population. Thus, viral infection appears to select for distal V_H_ genes, but if secondary rearrangement is playing a role in lengthening CDR-H3, it appears to be doing so only for the ChI Abs.

Many of our conclusions should be interpreted with caution, given that they are based on a limited dataset that may be biased in several ways. For example, there was significant bias related to Ag specificity for several categories of MAb; many of the anti-HIV MAbs were against the gp120 CD4bs, and were obtained *via* phage-displayed Ab libraries; most of the SAD MAbs were against the non-protein Ags DNA and phospholipid/cardiolipin; whereas most of the AcI MAbs were against streptococcal capsular polysaccharide and rotavirus. Another potential bias is related to the limited number of SADs we studied, with the preponderance being SLE and anti-phospholipid syndrome. Since we are studying MAbs, it is important to realize that particular antibodies are often selected for further study based on characteristics such as strength of binding, isotype or epitope, and thus the data set is not random with respect to these parameters. This is one reason why we adopted a conservative approach, and emphasize those results that satisfy a Bonferroni-adjusted p value based on the total number of tests conducted in Table 1. A larger dataset, indexed by disease and clinical condition, would overcome many of these potential biases. One roadblock to such a compilation is that many researchers do not routinely submit expressed sequences to public databases; this will become especially critical as high-throughput methods are employed to survey large sets of disease-specific MAbs.

This analysis of 427 Ag-specific MAbs should directly inform vaccine research. For example, the result that long CDR-H3s are associated with chronic and persistent Ag and anti-protein Abs motivates several questions. Are Abs bearing long CDR-H3s present at the beginning of an immune response, or do they “evolve” over time? If they accumulate over time, then are they directly selected from a pre-existing minor compartment within the naïve B-cell populations, do they comprise a specially recruited B-cell subset, and/or do they evolve by secondary processes (*e.g*., V_H_-gene replacement, DNA insertion, or gene conversion)? All of the bNt MAbs against HIV are heavily mutated and five of the eight have long CDR-H3s. This line of reasoning raises the possibility that long CDR-H3s are not required to bind conserved epitopes on the HIV-1 envelope, but arise instead through processes that come into play during long-term persistence of protein Ag and viral escape. If so, an effective HIV vaccine may produce bNt Abs *via* "normal" immunization processes, by virtue of enhancing the immunogenicity of Nt sites on Env. Given this scenario, it remains unknown if Abs bearing the features of acute antiviral Abs (which we expect to be similar to the features of Abs elicited by a traditional vaccine) can act as bNt Abs. While bNt Abs have yet to be elicited by vaccines meant to mimic the epitopes on Env that mediate neutralization by the bNt MAbs, that should not be taken as evidence that they cannot be so produced. Our results indicate that HIV vaccine research should continue to follow “reverse vaccinology” approaches [56] that attempt to make the sites recognized by the bNt MAbs immunodominant [57], [58]. Progress in this approach has recently been observed with an influenza vaccine that elicts broadly protective Abs [59]. Conversely, it is also possible, but not proven, that “chronic” type Abs bearing the features of the bNt MAbs will be required for broad neutralization. If this is the case, then research into the cellular and genetic origins of such Abs is required. Thus our second recommendation is for research efforts to be expanded in this area, with the goal of developing vaccination strategies that stimulate key features of these chronic processes, and in so doing, elicit bNt Abs.

## Materials and Methods

### Sequence database

Heavy chain sequences of expressed MAbs were retrieved from the IMGT/LIGM-DB on-line database (http://imgt.cines.fr/), from the literature, and from direct contacts with researchers (see Table S1). Our goal was to collect V_H_ sequences for all of the available human HIV MAbs. The Ag targets of the HIV MAbs included gp120, its CD4 binding site (CD4bs) and CD4 inducible site (CD4i), the gp120 V3 loop, gp41, Rev, Tat, p24, and p25. This MAb dataset was expanded to include human MAbs associated with other chronic infections (the ChI MAbs), including those against Epstein Barr virus, hepatitis B and C virus, herpes simplex virus and human cytomegalovirus. (Note that all of these MAbs are from viral infections.) For comparison, a similar group of MAbs from Systemic Autoimmune Disease (SAD) was assembled, including from systemic lupus erythematosus (SLE), anti-phospholipid syndrome, mixed connective tissue disease, rheumatoid arthritis, Sjögren's disease, and cold agglutin disease, and against the Ags, cardiolipin (serum dependent and serum independent), phospholipids, DNA, beta-2-glycoprotein, Sm ribonucleoprotiens, myelin basic protein, myelin-associated glycoprotein, achetylcholine receptor, Ro/SSA and La/SSB. We concentrated on SAD MAbs based on the hypothesis that the bNt anti-HIV MAbs were derived from autoAb/autoreactive precursors [19], [20], [42]. In addition, V_H_ sequences were collected for MAbs associated with acute infections (*Pseudomonas aeruginosa*, rotavirus, *Pneumococcus pneumoniae*, Ebola virus, *Neisseria meningitidis*, hepatitis A virus), and from vaccinated individuals (*Haemophilus influenzae* Type b conjugate vaccine, 23-valent pneumococcal polysaccharide vaccine, *Streptococcus pneumonia*, tetanus toxoid, hepatitis B surface Ag), reported as the AcI MAbs. For each MAb, we attempted to obtain information on its isotype, Ag specificity, the methods used to obtain it (*e.g.*, phage display, B-cell sorting, *etc.*), clinical data on the source-subject, and the bibliographic reference and GenBank accession number for the original MAb sequence. This information was entered into an Excel database by hand.

### Sequence analysis

Nucleotide sequences were analyzed using a recent version of JoinSolver (http://joinsolver.niams.nih.gov/index.htm; [60]), which provides the closest-matched V_H_, D_H_ and J_H_ genes, determines the limits of the CDR-H3 region, the length (in amino acids) of CDR-H3 region, the contributions of P and N nucleotides at both the V-D and D-J junctions, and the number of SMs in the MAbs relative to the predicted germline genes (this number is defined as of the number of base pair substitutions relative to a predicted germline gene). These results, including nucleotide sequence for CDR-H3 region, were also entered into the Excel database. For a few HIV MAbs, only CDR-H3 length, and not the expressed V_H_ sequence, was available (*e.g.*, Ditzel *et al.* [61]; see Table S1). Results from JoinSolver were compared to those produced by the V-QUEST and JunctionAnalysis algorithms of the IMGT system [62], which also analyzes V_H_ sequences for gene usage and somatic mutations. In addition, assignments of each MAb to predicted germline V_H_, D_H_ and J_H_ genes were confirmed visually. Results from IMGT and JoinSolver differed systematically. For example, the size of the region of CDR-H3 contributed by the germline D_H_ gene was consistently estimated to be greater using IMGT V-QUEST. This result can be explained by the fact that standard parameters for V-QUEST allow more mutations in the D_H_-gene core. However, differences between classes of MAbs were similar whether the comparisons were calculated using V-QUEST or JoinSolver, and these relative differences (*e.g*., the average CDR-H3 length of self *vs.* non-self MAbs) are the important parameters in our study.

JoinSolver results were used to screen for clonal expansions, which were identified as those Abs that used the same sets of V_H_, D_H_ and J_H_ regions with similar patterns of N and P nucleotides. A single, randomly chosen MAb was retained to represent each clonally-expanded set. Two recently reported bNt MAbs [7] are expansions of the same B-cell lineage, so we randomly selected one, PG16, for analysis; taking the same approach we selected VRC01 from the set of two bNt MAbs reported by Wu *et al*. [9]. Thus, the final set that was statistically analyzed does not include all reported HIV MAbs, but only those representing independent clonal lineages (see below, and Tables S1 and S2).

In summary, the entire database consists of over 700 MAbs (Table S1), which underwent two screens to produce the final dataset (Table S2) for analysis of CDR-H3 length, SM and gene usage. In the first screen, each MAb had to have a specified Ag, and to be associated with a particular immune response. In the second screen, clones from the same clonal expansion were deleted from the dataset, resulting in a 427-MAb dataset comprising 227 ChI MAbs (including 193 HIV MAbs), 87 SAD MAbs and 113 AcI MAbs, which was exported to SAS (Rel. 8.2, 2001; SAS Institute Inc., Cary, NC) for statistical analysis. Of these 427 MAbs, 318 were identified as to IgM or IgG; 90% of these were IgG (Table S2).

### Statistical analysis

For all MAb categories, PROC UNIVARIATE (SAS) was used to test the distributions of CDR-H3 length, total V_H_-gene mutations, distance of predicted V_H_ gene used in the MAb relative to V_H_6-1, the V-gene most proximal to the D_H_ region (**V_H_-distance**), and distance of predicted J_H_ gene from J_H_6, the J_H_ gene most distal to the D_H_ region (**J_H_-distance**), against the normal distribution. Most of the distributions were non-normal, even after log transformation, so a non-parametric Kruskall-Wallis Test was used to test for differences among sets of MAbs (PROC NPAR1WAY, SAS). To avoid zero values, the natural log of (3*CDR-H3 length in aa + 0.1) was used in tests for differences in CDR-H3 length. All results from the non-parametric tests were compared to one-way ANOVA (PROC GLM, SAS), and in all cases the results were similar in terms of levels of significance. When more than two categories were compared, (*i.e.,* comparisons among ChI, AcI and SAD MAbs), Tukey *a posteriori* tests were used to determine what groups were statistically different, and these different groups were denoted by different letters (PROC GLM, SAS). In **Table 1** we present the p values for the main statistical tests of this study. This Table reports 9 hypothesis tests for each of CDR-H3 length, number of SMs and V_H_-distance, for a total of 27 tests; therefore, to be conservative, all tests that passed a Bonferroni-corrected P value of 0.05/27 = 0.0018 were highlighted in bold. Given the many confounding factors in this data base, these probability values should be interpreted as indicators of strong differences among categories rather than strictly interpreted statistical tests (see Discussion). Distributions of CDR-H3 length for CD4bs, CD4i, V3 loop, and anti-gp41 MAbs presented in Figure 2 were tested for heterogeneity by χ^2^. J_H_-distance did not vary among MAb categories and is not reported. The difficulty of assigning germline D_H_ genes to expressed Ab sequences, especially for highly mutated HIV MAbs, precluded a comprehensive analysis of D_H_ gene usage or the number of P and N nucleotides.

## Supporting Information

Figure S1 **V_H_ gene family usage in anti-protein and non anti-protein MAbs for 3 disease conditions.** See Table 3 for sample sizes; there is only 1 ChI Mab that is not anti-protein. MAbs utilizing V_H_1 family were separated into those using V_H_1-69 and others.
(TIF)

Table S1 Database of antigen-specific expressed human MAbs.
(XLS)

Table S2 Expressed MAbs analyzed for CDR-H3 length, somatic mutations, and gene usage.
(XLS)
