# Supplementary figures and images for: Comparison of Antibody Repertoires Produced by HIV-1 Infection, Other Chronic and Acute Infections, and Systemic Autoimmune Disease
Source: PLoS One. 2011 Mar 30;6(3):e16857. doi: 10.1371/journal.pone.0016857 (PMC3068138; doi:10.1371/journal.pone.0016857)

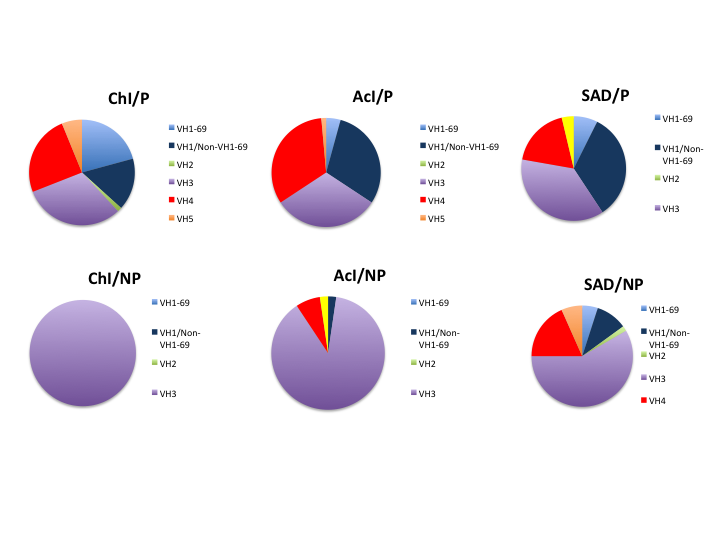

Supplement: Figure S1 — VH gene family usage in anti-protein and non anti-protein MAbs for 3 disease conditions. See Table 3 for sample sizes; there is only 1 ChI Mab that is not anti-protein. MAbs utilizing VH1 family were separated into those using VH1-69 and others. (TIF) [file pone.0016857.s001.tif]
